# Using Spatial Scan Statistics and Geographic Information Systems to Detect Monthly Human Mobility Clusters and Analyze Cluster Area Characteristics

**DOI:** 10.31662/jmaj.2023-0208

**Published:** 2024-06-10

**Authors:** Ryo Horiike, Tomoya Itatani, Hisao Nakai, Daisuke Nishioka, Aoi Kataoka, Yuri Ito

**Affiliations:** 1 Department of Public Health Nursing, Osaka Medical and Pharmaceutical University, Osaka, Japan; 2 Division of Home Care Nursing, Department of Fundamental and Community Nursing Science, School of Nursing, Faculty of Medicine, University of Miyazaki, Miyazaki, Japan; 3 Faculty of Nursing, University of Kochi, Kochi, Japan; 4 Department of Medical Statistics, Research & Development Center, Osaka Medical and Pharmaceutical University, Osaka, Japan; 5 Division of Molecular Epidemiology, Department of Future Medicine Sciences, Kobe University Graduate School of Medicine, Hyogo, Japan

**Keywords:** spatial scan statistics, infection disease, geographic information systems, COVID-19, evidence-based policymaking

## Abstract

**Introduction:**

This study evaluated the detection of monthly human mobility clusters and characteristics of cluster areas before the coronavirus disease 2019 (COVID-19) outbreak using spatial epidemiological methods, namely, spatial scan statistics and geographic information systems (GIS).

**Methods:**

The research area covers approximately 10.3 km^2^, with a population of about 350,000 people. Analysis was conducted using open data, with the exception of one dataset. Human mobility and population data were used on a 1-km mesh scale, and business location data were used to examine the area characteristics. Data from January to December 2019 were utilized to detect human mobility clusters before the COVID-19 pandemic. Spatial scan statistics were performed using SaTScan to calculate relative risk (RR). The detected clusters and other data were visualized in QGIS to explore the features of the cluster areas.

**Results:**

Spatial scan statistics identified 33 clusters. The detailed analysis focused on clusters with an RR exceeding 1.5. Meshes with an RR over 1.5 included one with clusters for 1 year which is identified in all months of the year, one with clusters for 9 months, three with clusters for 6 months, three with clusters for 3 months, and four with clusters for 1 month. September had the highest number of clusters (eight), followed by April and November (seven each). The remaining months had five or six clusters. Characteristically, the cluster areas included the vicinity of railway stations, densely populated business areas, ball game fields, and large-scale construction sites.

**Conclusions:**

Statistical analysis of human mobility clusters using open data and open-source tools is crucial for the advancement of evidence-based policymaking based on scientific facts, not only for novel infectious diseases but also for existing ones, such as influenza.

## Introduction

Coronavirus disease 2019 (COVID-19) is an infectious disease that has affected over 760 million people worldwide, resulting in more than 6.8 million deaths ^(1)^. Various strategies have been employed to combat the COVID-19 pandemic, including nonpharmaceutical interventions (NPIs), such as effective communication strategies and governmental support; strict measures, such as lockdowns; and pharmaceutical interventions, such as vaccines and antiviral drugs ^(2)^. Since the early stages of the pandemic outbreak, NPIs delayed the spread of the infection as effectively as strict solutions. NPIs typically involve measures such as social distancing and cancellation of small-scale gatherings ^(3)^, primarily implemented by local health departments. However, restrictions imposed by local governments can infringe human rights ^(4)^. Therefore, nonrestrictive and effective measures are necessary. Moreover, infectious disease policies should be grounded in scientific facts to form the basis for evidence-based policymaking (EBPM).

Avoiding infectious disease clusters through NPIs is crucial to containing infection and death rates. Spatial epidemiology and spatial scan statistics (SSS), including the examination of disease clusters, have been employed in the analysis of cancer incidence rates, healthcare sectors, and COVID-19 clusters ^(5), (6), (7), (8), (9)^. To date, the data utilized to develop COVID-19 countermeasures have been derived from the examination of disease clusters after the outbreak. Analyzing the characteristics of human mobility clusters using data from periods without outbreaks is essential to rapidly manage emerging infectious diseases and prepare business continuity plans for health administrations. In this study, “human mobility cluster” refers to the aggregation of groups in an area resulting from human movement and is not meant to denote the aggregation of infectious disease patients, which is commonly used in the context of infectious diseases ^(10), (11), (12)^. In Japan, human flow data have been used to support NPIs, such as social distancing ^(13)^, and statistical identification of human mobility clusters is necessary because dense human gatherings pose a risk of COVID-19 cluster formation.

This study aimed to detect human mobility clusters before the COVID-19 pandemic using SSS and elucidate the characteristics of cluster areas, thereby facilitating the implementation of NPIs.

## Materials and Methods

### Data

This study used open data available online, with one exception (business location information). Boundary data for the target region, using administrative district data, were obtained from the National Land Numerical Information site of the Ministry of Land, Infrastructure, Transport and Tourism ^(14)^. Residential population data, using future population data with a 1-km mesh (H30 National Bureau Estimates), were also obtained from the same site ^(15)^. The future estimated population was calculated by the National Institute of Population and Social Security Research using the 1-km mesh format based on the 2018 census results every 5 years from 2020 to 2050 ^(16)^. Mesh data, a digitalized map format for various statistical information ^(17)^, were used, with each mesh being a 1-km square dividing the area. However, as no data were available for 2019, the 2020 total estimated population was used. Human flow data were obtained from the nationwide human flow open data of the G Spatial Information Center (1-km mesh) ^(18)^. The resident population was based on GPS data collected from smartphones using Agoop SDK ^(19)^. The average number of people per day during 1 month was calculated based on the converted population value, and the data were available from January 2019 on a monthly basis. Monthly data between January and December 2019 were selected to exclude the impact of COVID-19. Comprehensive data were obtained by selecting the full-day data for each month. As railways affect human flow, railway station and line data were obtained from the National Land Numerical Information site ^(20)^.

Business location information was purchased from Zenrin Co., Ltd., which sells corporate search data. These data combine information, including location data, on approximately 6 million corporations in Japan, encompassing a wide range of companies and organizations ^(21)^. In this study, the data were classified and mapped into five categories: eateries, customer attraction stores, offices, retail stores, and medical and care facilities.

**Figure 1.**
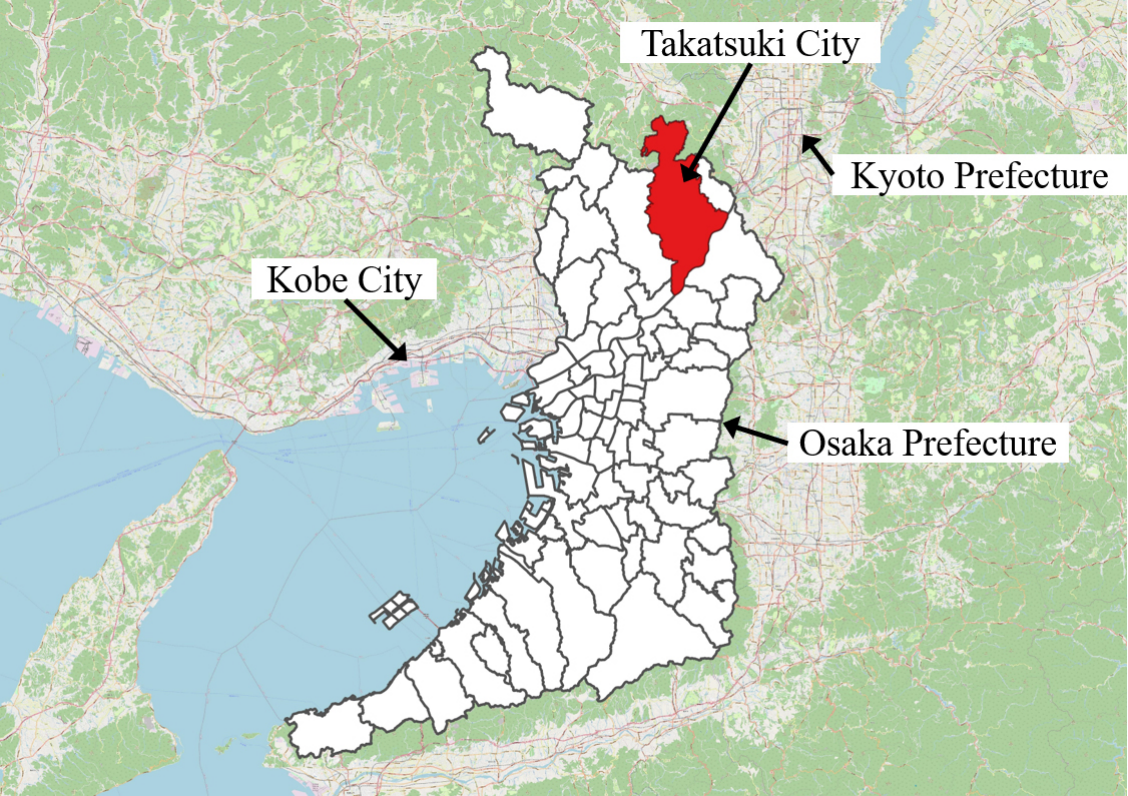
Takatsuki City (indicated in red). ^Ⓒ^OpenStreetMap contributors ^(38)^

Figure 1 shows the geographical location of Takatsuki City in Japan, where the analysis was conducted. Takatsuki City is a municipality located in Osaka Prefecture, Western Japan, between Kyoto and Osaka. The northern part of the city is mountainous, featuring scenic tourist spots along highways, whereas the southern part is urban, with a mix of redeveloped high-rise apartments and traditional houses. Two railway companies operate in the city, with a major commercial area centered around the railway station in the city center. The population is approximately 350,000, with children below 14 years old, people of productive age (15-64 years old), and older adults (65 years old or older) accounting for 21%, 57%, and 28% of the total population ^(22)^.

### Mapping

To examine the geographical distribution of the future estimated population and human flow data, QGIS (version 3.28.3) was used. QGIS, an official project of the Open Source Geospatial Foundation, is a user-friendly open-source geographic information system (GIS) ^(23)^. It has an intuitive UI and robust spatial analysis capabilities and is continually upgraded with numerous additional features through plugins developed by contributors worldwide. The downloaded data were inputted into QGIS for mapping, enabling data visualization and examination of the geographical distribution of clusters identified through scan statistics. Furthermore, the characteristics of businesses located in the detected cluster areas were obtained using corporate search data. To understand the geographical trends, aerial photographs from the Geospatial Information Authority of Japan’s Geospatial Information Tiles were used as background maps ^(24)^.

### Spatial scan statistics (SSS) using the poisson distribution

SaTScan™ (version 10.1, 64 bits) was used to detect clusters ^(25)^. SaTScan was developed in 1997 by Professor Kulldorff of Harvard Medical School. It is a free software capable of performing statistical analyses to detect clusters (disease agglomerations) in space or space-time ^(26)^. SSS involves the use of a window, referred to as a connected area, that could potentially be a cluster within a larger connected region. This circular window was continuously expanded and moved, and the window with the maximum likelihood ratio, as determined by a Monte Carlo probability simulation, was considered to be the most likely cluster ^(26), (27)^. If the observed values within a window are significantly higher than the expected values based on the Poisson distribution, this indicates the presence of a cluster. Conversely, it is also possible to detect clusters that are not statistically significant. Expected values can be calculated using the area population, and the degree of agglomeration is expressed as relative risk (RR). SSS has been applied in various fields beyond epidemiological research and is particularly effective in detecting hotspot clusters in suburban areas with low population densities. Thus, it is suitable for detecting clusters in areas such as the study area ^(28)^, with a densely populated southern area and mountainous northern area with a low population density.

The following SaTScan settings were used to conduct SSS (Table 1). The location ID in the coordinate file corresponded to the mesh ID, and the centroids of each mesh were calculated using the geometry tool in QGIS. The latitude and longitude were obtained using the function feature of the field calculator.

**Table 1.** Data and Settings for Conducting Spatial Scan Statistics.

Software settings	Spatial Scan Statistics
Case File	Population in 1-km mesh
Population File	Future population projection in 1-km mesh (total number of men and women in 2020)
Coordinate File	Latitude and longitude of the 1-km mesh center of the gravity point
Probability Model	Discrete Poisson
Spatial Window Shape	Circular
Maximum Spatial Cluster Size	50% of the population at risk
Maximum Monte Carlo Permutations	999
Criteria for Reporting Secondary Clusters	No geographical overlap
*P*-value	*P* < 0.05

Furthermore, the space-time scan statistic (STSS) can be performed using SaTScan if the concept of time is included ^(29)^. However, STSS is suitable for examining when the largest clusters occur within a specific period, whereas SSS is more appropriate for detecting monthly clusters and identifying their characteristics ^(30)^, which was the focus of this study. Therefore, this study employed SSS for cluster detection.

## Results

**Figure 2.**
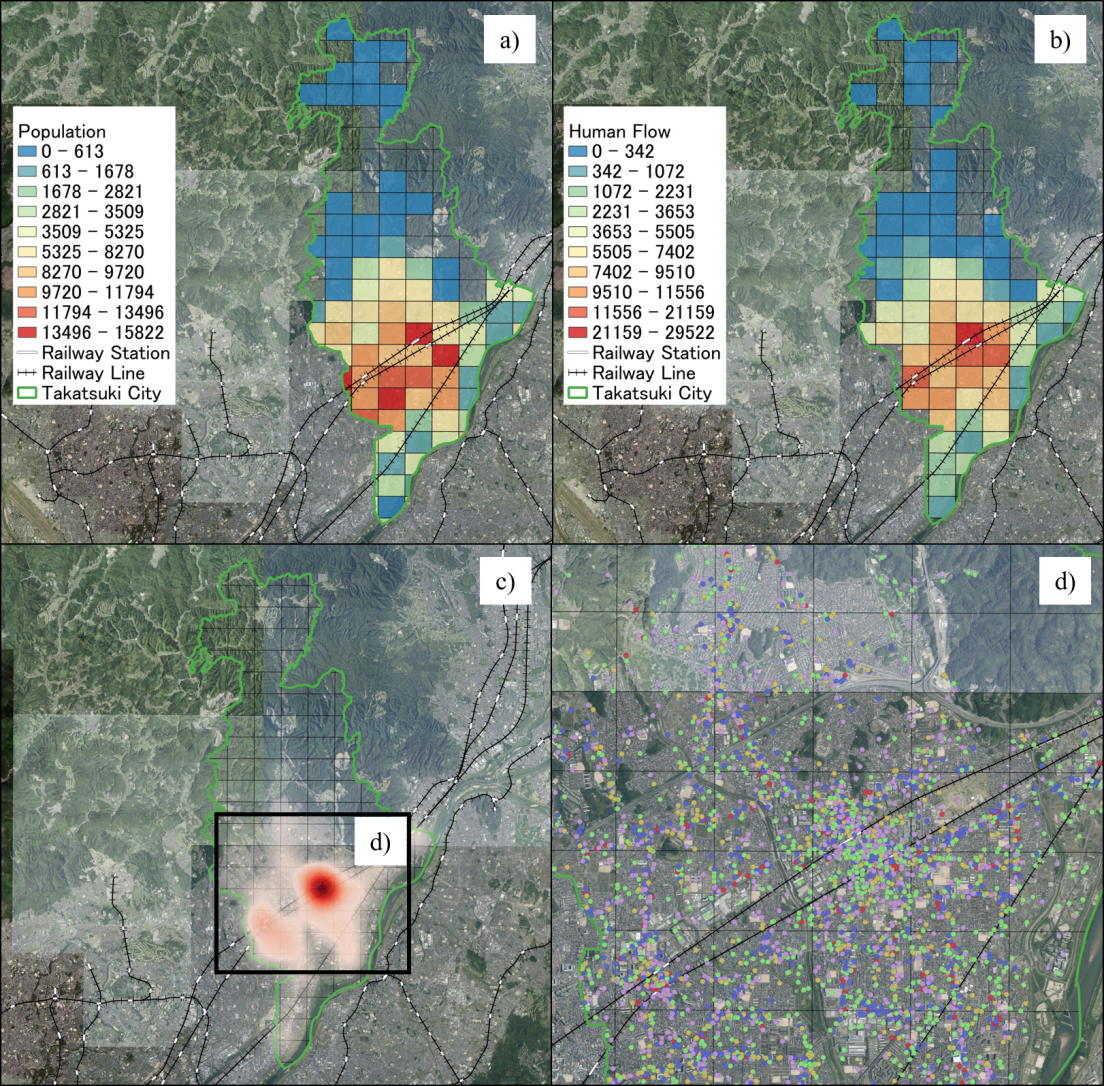
(a) Residential population per mesh (blue = few residents; red = many residents). (b) Human flow per mesh for January 2019 (blue = few residents; red = many residents). (c) Heatmap based on corporate search data demonstrating the distribution of business locations. (d) Business locations in the southern part of the city, where businesses are most densely situated (red: eateries; pink: customer attraction stores; blue: retail stores; green: offices; yellow: medical and care facilities). Each side of all black squares is 1 km. GSI Tile (National Latest Photo [Seamless]) provided by GSI is used as a background map.

Figure 2 shows the geographical distribution of the residential population, human flow, and business locations in Takatsuki City. The residential population of Takatsuki City in 2020 was concentrated in the southern part of the city, particularly along the railway lines. Contrarily, the northern part, which is mountainous, had a smaller residential population with some meshes containing no residents. The human flow data for January 2019 showed a similar trend to that of the residential population, with a higher concentration of people around the four railway stations in the city. In addition, the heatmap showed a concentration of business locations around railway stations in the southern part of the city. Expanding the area where businesses are concentrated and mapping their locations enable a detailed understanding of the geographical distribution trends. This instance examined only the industry type; however, the subsequent analysis utilized data contained in the business location information, including business names, addresses, detailed industry types, and latitudes and longitudes (only location information is shown Figure 2d) due to data usage agreement terms).

### Cluster detection using spatial scan statistics (SSS)

**Figure 3.**
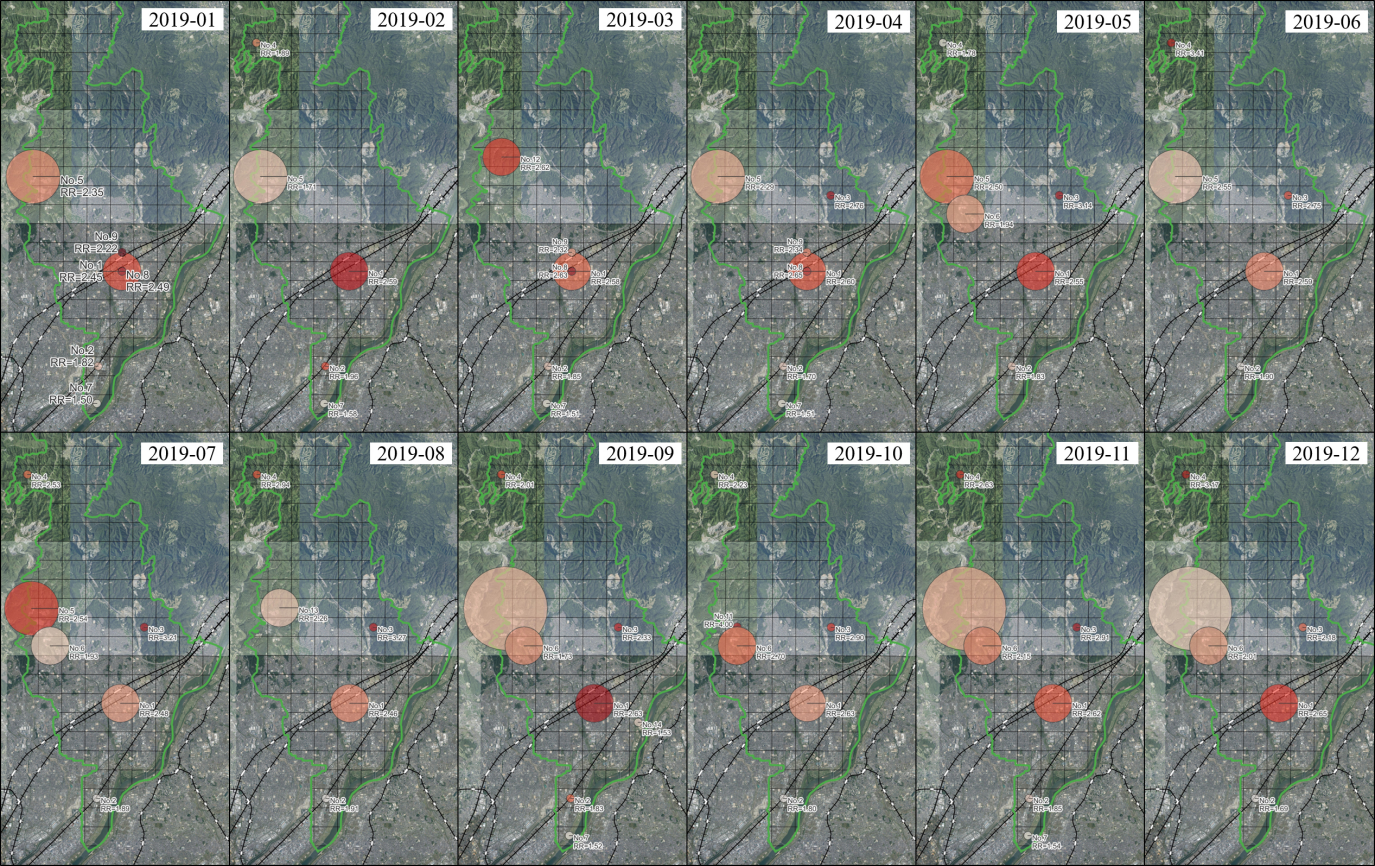
Geographical location and relative risk of clusters detected using spatial scan statistics (SSS) in each month between January and December 2019. GSI Tile (National Latest Photo [Seamless]) provided by GSI is used as a background map.

SSS revealed that 33 meshes had significant clusters detected in at least 1 month of 2019. Some meshes showed significant clusters throughout the year, whereas others were identified as clusters only during specific months. Clusters with an RR below 1.5 exhibited low monthly variation and consistently remained below 1.5. Therefore, 14 meshes with an RR exceeding 1.5 were selected for a detailed analysis because they represented areas with a high risk of human gathering and significant monthly variability, potentially indicating the occurrence of seasonal events (Table 2 and Figure 3). Meshes with an RR above 1.5 included two that showed clusters throughout the year (1 and 2), two for 9 months (3 and 4), three for 6 months (5, 6, and 7), three for 3 months (8, 9, and 10), and four for 1 month (11, 12, 13, and 14). September had the most clusters (eight), followed by April and November (seven each), whereas the remaining months had five or six clusters.

**Table 2.** Detected Clusters with a Relative Risk Above 1.5.

**No.**	**Mesh ID**	**2019-01**				**2019-02**				**2019-03**				**2019-04**			
		**Observation**	**Expected** **Case**	**Population**	**RR**	**Observation** **Case**	**Expected** **Case**	**Population**	**RR**	**Observation** **Case**	**Expected** **Case**	**Population**	**RR**	**Observation** **Case**	**Expected** **Case**	**Population**	**RR**
1	52352419, 52352429	50,681	22,838.28	23,962	2.45	52,411	22,499.00	23,962	2.59	52,389	22,564.52	23,962	2.58	52,982	22,684.52	23,962	2.6
2	52351468	1,873	1,032.25	1,083	1.82	1,983	1,016.92	1,083	1.96	1,879	1,019.88	1,083	1.85	1,743	1,025.30	1,083	1.7
3	52352550													219	79.39	84	2.76
4	52353435					52	27.55	29	1.89								
5	52352465, 52352466 52352456, 52352476	1,011	431.10	452	2.35	726	424.70	452	1.71					981	428.20	452	2.29
6	52352446, 52352456																
7	52351448	512	342.00	359	1.50	532	336.92	359	1.58	510	337.90	359	1.51	512	339.69	359	1.51
8	52352419	21,159	8,856.15	9,292	2.49					22,052	8,749.99	9,292	2.63	22,268	8,796.52	9,292	2.65
9	52352429	29,522	13,982.13	14,670	2.22					30,337	13,814.53	14,670	2.32	30,714	13,888.00	14,670	2.34
10	52352465, 52352466 52352456, 52352476 52352446																
11	52352456																
12	52352476, 52352486									157	59.86	64	2.62				
13	52352466, 52352465 52352476																
14	52352501																
**No.**	**Mesh ID**	**2019-05**				**2019-06**				**2019-07**				**2019-08**			
		**Observation** **Case**	**Expected** **Case**	**Population**	**RR**	**Observation** **Case**	**Expected** **Case**	**Population**	**RR**	**Observation** **Case**	**Expected** **Case**	**Population**	**RR**	**Observation** **Case**	**Expected** **Case**	**Population**	**RR**
1	52352419, 52352429	52,698	22,934.34	23,962	2.55	52,796	22,787.52	23,962	2.59	50,767	22,633.56	23,962	2.48	50,143	22,523.75	23,962	2.46
2	52351468	1,892	1,036.60	1,083	1.83	1,946	1,029.96	1,083	1.9	1,925	1,023.00	1,083	1.89	1,941	1,018.04	1,083	1.91
3	52352550	252	80.27	84	3.14	219	79.75	84	2.75	254	79.22	84	3.21	258	78.83	84	3.27
4	52353435	50	28.08	29	1.78	95	27.90	29	3.41	70	27.71	29	2.53	81	27.58	29	2.94
5	52352465, 52352466 52352456, 52352476	1,080	432.92	452	2.5	1,096	430.15	452	2.55	1,085	427.24	452	2.54				
6	52352446, 52352456	1,279	659.04	689	1.94					1,251	650.40	689	1.93				
7	52351448																
8	52352419																
9	52352429																
10	52352465, 52352466 52352456, 52352476 52352446																
11	52352456																
12	52352476, 52352486																
13	52352466, 52352465 52352476													351	155.07	165	2.26
14	52352501																
**No.**	**Mesh ID**	**2019-09**				**2019-10**				**2019-11**				**2019-12**			
		**Observation** **Case**	**Expected** **Case**	**Population**	**RR**	**Observation** **Case**	**Expected** **Case**	**Population**	**RR**	**Observation** **Case**	**Expected** **Case**	**Population**	**RR**	**Observation** **Case**	**Expected** **Case**	**Population**	**RR**
1	52352419, 52352429	53,489	22,702.64	23,962	2.63	53,578	22,747.16	23,962	2.63	53,169	22,676.04	23,962	2.62	55,021	23,201.01	23,962	2.65
2	52351468	1,868	1,026.12	1,083	1.83	1,841	1,028.14	1,083	1.8	1,891	1,024.92	1,083	1.85	1,770	1,048.65	1,083	1.69
3	52352550	185	79.46	84	2.33	231	79.61	84	2.9	231	79.36	84	2.91	177	81.20	84	2.18
4	52353435	56	27.80	29	2.01	62	27.85	29	2.23	73	27.76	29	2.63	90	28.41	29	3.17
5	52352465, 52352466 52352456, 52352476																
6	52352446, 52352456	1,129	652.38	689	1.73	1,756	653.66	689	2.7	1,400	651.62	689	2.15	1,340	666.70	689	2.01
7	52351448	517	339.96	359	1.52					521	339.57	359	1.54				
8	52352419																
9	52352429																
10	52352465, 52352466 52352456, 52352476 52352446	1,356	808.68	854	1.68					1647	807.74	854	2.04	1,580	826.44	854	1.92
11	52352456					1,089	272.77	287	4								
12	52352476, 52352486																
13	52352466, 52352465 52352476																
14	52352501	1,125	737.71	779	1.53												

Notes. Mesh ID, unique number of the 1-km square grid dividing the area; Observation Case, number of people in the mesh; Expected Case, expected number of people as calculated using spatial scan statistics; Population, residential population in the mesh; RR, relative risk calculated using the spatial scan statistics. All clusters were considered statistically significant at *P* < 0.01.

### Characteristics of cluster areas

Factors potentially contributing to high risk in the detected cluster areas were identified using business location information. Cluster 1 was detected throughout the year and consistently exhibited a high monthly RR > 2.4. Cluster 1 was located in a hub for public transportation with stations of various railway companies and a high concentration of eateries and offices, which matched the heatmap results. Cluster 3 was located in the central-eastern part and formed in April 2019. A major highway expansion project was identified at this location. Clusters 5, 6, 10, and 13 shared common features, such as comprehensive sports parks, baseball fields, soccer fields, and tennis courts. Cluster 7, which was detected from January to April, September, and November, was identified as a golf course. Cluster 11 had a high RR (RR = 4) only in October and included a valley known for beautiful autumn leaves and hot springs. Similarly, Cluster 12, which was detected only in March, encompasses a mountaintop well known for cherry blossoms.

## Discussion

This study identified monthly human mobility clusters and their characteristics before the COVID-19 pandemic using SSS, GIS, and integrated business location data. The results indicated that human mobility clusters were associated with areas central to public transportation, commercial areas with a high concentration of eateries and offices, construction sites, comprehensive sports parks, and ball game fields. Seasonal variations were also observed.

The use of GIS to represent multiple data types on a single map is crucial. This enables a realistic representation of physical spaces based on geographical spatial information by combining data on population dynamics, human mobility, transportation, and business locations. This approach is crucial for public health professionals, including health officers, for community diagnosis and surveillance, not only during outbreaks but also in regular times ^(31)^.

Mapping of SSS results in GIS promotes a common understanding among epidemiologists, public health experts, and the general public. SaTScan outputs cluster information in widely used GIS formats, such as Shapefiles and KML files for Google Earth ^(32)^.

This study conducted SSS on a computer; however, cloud implementation could allow for on-demand execution, reduce processing time, and benefit health authorities when using large datasets ^(33)^.

The detected cluster areas were confirmed using business location information. However, some clusters, such as 4 and 7, were detected in areas without corresponding business locations, indicating that some clusters may be transit points. For instance, a large golf course was located beyond Cluster 4. Road information and data from neighboring municipalities could provide further insight into cluster characteristics ^(34)^.

In addition, local interviews could be valuable, as local customs and festivals are potential cluster sources ^(35)^. However, these data are often not openly available. Combining GIS with interviews in spatial epidemiology can validate the spatial analysis results ^(36)^.

Evidence-based policy decisions are preferred, particularly for public institutions that implement policies restricting human rights or require substantial budgets. This study identified areas prone to human mobility clusters and suggested areas where interventions should be seasonally intensified or relaxed. During the COVID-19 response, European countries initially justified lockdowns based on science but later prioritized economic values and voter opinions over scientific advice ^(37)^. To implement NPIs that are reasonable for human rights, movement and similar measures can be restricted to the smallest possible population within the minimum necessary areas to suppress the spread of infectious diseases. In addition, by identifying outbreak-prone areas based on the flow of people and characteristics of regions during normal times, it is feasible to issue preemptive warnings as NPIs based on data before imposing movement restrictions. As a result, if the outbreak is suppressed, it will be possible to minimize the need for movement restrictions and other measures, thereby protecting human rights while controlling the spread of infectious diseases.

Promoting EBPM based on open data and open-source science facts is imperative, not only for new infectious diseases but also for existing ones, such as influenza and RSV ^(3)^. To utilize the results of this study for the implementation of NPIs, it is important for municipalities or prefectural governments to estimate baseline data during normal times. Normal times refer to periods before the arrival of new infectious diseases or when diseases such as influenza are not in an outbreak phase. By continuously understanding the baseline data of people’s movements, it is believed that anomalies can be detected and compare with the baseline when an infectious disease outbreak occurs. In addition, attempting to calculate the baseline after an outbreak of an infectious disease could influence the time and human resources required to implement outbreak suppression policies. Therefore, it is necessary to automate based on the results of this study using a system, thereby reducing the burden on public health officials.

The reason for this is that the results of this study can flexibly adapt to the differences in transmission patterns, groups susceptible to infection, or age groups prone to severe illness for each infectious disease. Depending on whether the infection is spread through contact or airborne transmission, the size of the area for detecting human mobility clusters can vary. Moreover, the data on human flow includes information such as age, gender, and starting points. Although this study analyzed the data for the entire population, it is possible to limit the analysis only to data for the elderly or for children based on the characteristics of each infectious disease. This indicates the possibility of developing customized outbreak suppression policies tailored to individual infectious diseases. Furthermore, by accumulating these insights, when an unknown infectious disease emerges, the best NPIs can be selected by processing the vast amount of accumulated data with generative AI.

The universality of this methodology allows similar analyses worldwide. All data utilized, except one source, were open. SaTScan and QGIS are open-source and freely available software packages for the implementation of SSS and GIS ^(38)^. Business location data, although not open, can be substituted with open-source alternatives, such as OpenStreetMap or free aerial and satellite images provided by national geospatial authorities ^(39)^.

SSS in SaTS can detect circular clusters; thus, noncircular clusters may be overlooked, and low-risk areas may be included in the results ^(40)^. Noncircular clusters can be detected using software such as FleXScan; however, this software requires more computational resources ^(41)^.

This study successfully employed spatial epidemiological methods, SSS, and GIS to detect human mobility clusters and analyze cluster area characteristics before the COVID-19 pandemic.

## Article Information

### Conflicts of Interest

None

### Sources of Funding

This study was supported by the JSPS Grant-in-Aid for Research Activity Start-up (KAKENHI; Grant Number JP 22K21188).

### Acknowledgement

We would like to thank Editage (www.editage.jp) for English language editing.

### Author Contributions

R.H. designed the study, main conceptual ideas, and proof outline; collected the data; aided in interpreting the results; and worked on the manuscript. T.I., H.N., D.N., A.K. and Y.I. supervised this study. All the authors discussed the results and commented on the manuscript.

### Approval by Institutional Review Board (IRB)

This study did not include personal information and utilized only existing data, making the need for such considerations unnecessary.
